# Quantification of aerobic determinants of performance in post-pubertal adolescent middle-distance runners

**DOI:** 10.1007/s00421-019-04175-w

**Published:** 2019-06-17

**Authors:** Richard C. Blagrove, Glyn Howatson, Charles R. Pedlar, Philip R. Hayes

**Affiliations:** 1 School of Sport, Exercise and Health Sciences, Loughborough University, Epinal Way, Loughborough, Leicestershire UK; 2 Department of Sport, Exercise and Rehabilitation, Northumbria University, Newcastle-upon-Tyne, UK; 3 Water Research Group, Northwest University, Potchefstroom, South Africa; 4 School of Sport, Health and Applied Science, St Mary’s University, Twickenham, UK; 5 Orreco Ltd, National University of Ireland Business Innovation Centre, Galway, Ireland; 6 Division of Surgery and Interventional Science, University College London, London, UK

**Keywords:** Running economy, Maximal oxygen uptake, Fractional utilization, Youth

## Abstract

**Purpose:**

The use of oxygen cost ($\dot{O}$_aero_) parameters to predict endurance performance has recently been criticized. Instead, it is suggested that aerobic energy cost ($\dot{E}_{\text{aero}}$) provides greater validity; however, a comparison of these quantification methods has not previously been made.

**Methods:**

Fifty-six male (*n* = 34) and female (*n* = 22) competitive adolescent (17 ± 1 years) middle-distance runners participated in a sub-maximal and maximal incremental treadmill test. Running economy (RE) was measured at the speed corresponding to lactate turnpoint, and the three speeds prior. Maximal oxygen uptake ($\dot{V}$O_2_max), speed at $\dot{V}$O_2_max and fraction of $\dot{V}$O_2_max utilized across a range of intensities, and speeds from 0.8, 1.5 and 3 km races were also quantified. RE and fractional utilization were calculated in units of $\dot{O}$_aero_ and $\dot{E}$_aero_.

**Results:**

Multiple linear regression models demonstrated no discernible difference in the predictive capability of RE, fractional utilization and $\dot{V}$O_2_max when expressed as $\dot{O}$_aero_ or $\dot{E}$_aero_ in both sexes. When plotted as a function of running speed, $\dot{O}$_aero_ displayed a stepwise decrease (*F* = 11.59, *p* < 0.001) whereas $\dot{E}$_aero_ exhibited a curvilinear response (*F* = 4.74, *p* = 0.015). Differences were also evident in the slopes plotted for %$\dot{V}$O_2_max and %$\dot{E}$_aero_max against running speed (*F* = 5.38, *p* = 0.021).

**Conclusions:**

Quantifying aerobic determinants of performance in units of $\dot{E}$_aero_ provides no greater validity compared to $\dot{O}$_aero_-based measurement. Although both $\dot{E}$_aero_ and $\dot{O}$_aero_ are sensitive to changes in speed, $\dot{E}$_aero_ provides the more valid reflection of the underlying metabolic cost of running. Physiologists should also be aware of the potential differences between expression of aerobic running intensity based upon %$\dot{V}$O_2_max compared to %$\dot{E}$_aero_max_._

## Introduction

Distance running performance is largely dependent upon aerobic factors, including maximal oxygen uptake ($\dot{V}$O_2_max), running economy (RE) and the fraction of $\dot{V}$O_2_max utilized over a given distance (Bassett and Howley 2000; Brandon 1995). Although the variability in distance running performance can largely be explained by $\dot{V}$O_2_max in heterogeneous groups of runners, RE and fractional utilization are better capable of predicting performance in runners homogenous for $\dot{V}$O_2_max (Conley and Krahenbuhl 1980). Specifically, in middle-distance events, a model that included $\dot{V}$O_2_max and RE, was capable of explaining 96% of the variance in performance in highly trained 800-m and 1500-m runners (Ingham et al. 2008). It has recently been suggested that expressing physiological parameters in terms of aerobic energy cost ($\dot{E}$_aero_) provides greater validity for quantifying exercise intensity compared to traditional oxygen cost ($\dot{O}$_aero_)-based measurements (Beck et al. 2018); however, these claims have not yet been fully examined with experimental data.

The expression of aerobic factors in units of $\dot{O}$_aero_ is limited because this measure does not account for differences in substrate utilization, which can vary substantially between runners operating at the same oxygen uptake ($\dot{V}$O_2_) (Brooks and Mercier 1994; Fletcher et al. 2009). It has been suggested that RE should, therefore, be quantified as Ė_aero_, which provides a more accurate reflection of the metabolic cost of exercise (Shaw et al. 2014). Previous reports have confirmed that $\dot{E}$_aero_ provides a more sensitive measure of RE compared to $\dot{O}$_aero_ across range of intensities in highly trained runners (Fletcher et al. 2009; Shaw et al. 2014); however, this has not yet been established in lesser trained populations of runners, such as adolescents. $\dot{E}$_aero_ appears to provide a more reliable measurement of RE compared to $\dot{O}$_aero_ in high-performing adolescent runners (Blagrove et al. 2017); however validity-related issues associated with these measures have not previously been scrutinized in this age group.

The physiological determinants of performance for adolescents are similar to those of adult runners. A number of investigations have confirmed that $\dot{V}$O_2max_ has a moderate–good correlation (*r* = 0.5–0.9) with performance over 1.5 km (Abe et al. 1998; Almarwaey et al. 2003), 3 km (Abe et al. 1998; Mahon et al. 1996; Unnithan et al. 1995), and 5 km (Abe et al. 1998; Cole et al. 2006; Cunningham 1990) in young (10–18 years) groups of runners. Measures of RE quantified in units of $\dot{V}$O_2_ also appear to be related to middle-distance performance (Almarwaey et al. 2003; Mayers and Gutin 1979; Unnithan et al. 1995). Additionally, speed at $\dot{V}$O_2max_ (s$\dot{V}$O_2max_) (Abe et al. 1998; Almarwaey et al. 2003; Cole et al. 2006; Cunningham 1990) and fractional utilization calculated in $\dot{V}$O_2_ terms have also been shown to significantly correlate with distance running performance in adolescents (Mahon et al. 1996; Unnithan et al. 1995). Despite these findings, for $\dot{E}$_aero_ to possess greater criterion validity compared to $\dot{O}$_aero_, it should be capable of predicting performance times with greater accuracy. This direct comparative analysis of two different approaches to quantifying aerobic-based determinants of performance has not previously been performed and is important for establishing validity of these metrics. Moreover, the method used to partition groups of young participants for differences in body size for variables such as $\dot{V}$O_2max_ and RE is also likely to influence findings (Eisenmann et al. 2001). Previous studies have normalized to body mass as a simple ratio (Abe et al. 1998; Almarwaey et al. 2003; Mahon et al. 1996; Unnithan et al. 1995); however, this is unlikely to appropriately partition out the confounding influence of body size (Loftin et al. 2016).

It has been proposed that fractional utilization expressed as the ratio between $\dot{E}$_aero_ and maximal aerobic energy expenditure ($\dot{E}$_aero_max) at lower intensities (respiratory exchange ratio (RER) < 1.0) provides a numerically lower relative aerobic intensity compared to fractional utilization quantified as %$\dot{V}$O_2_max (Beck et al. 2018). This has important implications for prescription of aerobic exercise intensity and for quantifying the physiological outcomes to training or nutritional interventions. Although this difference has been established in elite race walkers (Beck et al. 2018), no papers have attempted to compare these two approaches for other exercise modalities and sub-elite populations. Moreover, small differences in the predictive power of physiological determinants (expressed in $\dot{E}$_aero_ or $\dot{O}$_aero_ terms) on performance times may provide greater deterministic accuracy when combined as part of a multiple-factor regression model.

Consequently, the primary purpose of this study was to examine the relationship between physiological variables, quantified as both $\dot{E}$_aero_ and $\dot{O}$_aero_, and race performances in a group of competitive post-pubertal adolescent middle-distance runners. The secondary aims were to investigate the influence of running speed on RE quantified as both $\dot{O}$_aero_ and $\dot{E}$_aero_, and examine whether expressing relative aerobic intensity as %$\dot{V}$O_2_max and %$\dot{E}$_aero_max produces a different slope of values across a range of speeds. It was hypothesized that $\dot{E}$_aero_ would provide a more valid means of expressing important aerobic performance determinants compared to $\dot{O}$_aero_.

## Methods

### Participants

Following institutional level ethical approval and in accordance with the Helsinki declaration, 56 competitive male (*n* = 34) and female (*n* = 22) middle-distance (0.8–3 km) runners (15–18 years) volunteered to take part in this study. Participant descriptive statistics are displayed in Table 1. All participants possessed at least 2 years of distance running training and racing experience, were familiar with treadmill running and considered middle-distance running to be their main sport. Participants were informed of the requirements and risks associated with the study and thereafter signed consent to participate was obtained from a parent or guardian, or the participant themselves if > 18 years.

**Table 1:** Descriptive characteristics of the study participants

Measure	Males (*n* = 34)	Females (*n* = 22)
Age (year)	17 ± 1	17 ± 1
Stature (m)	1.76 ± 0.06	1.69 ± 0.06
Body mass (kg)	62.5 ± 6.4	52.7 ± 5.8
$\dot{V}$O_2_max (ml kg^−1^ min^−1^)	70.1 ± 7.2	61.1 ± 6.4
sLT (km h^−1^)	13.4 ± 1.5	11.7 ± 1.3
s$\dot{V}$O_2_max (km h^−1^)	19.2 ± 1.5	17.0 ± 1.5

$\dot{V}$*O*_*2*_*max* maximal oxygen uptake, *sLT* speed at lactate threshold, $s{\dot{V}}O$_*2*_*max* speed at $\dot{V}$O_2_max

### Procedure

All trials were conducted in the same laboratory under similar environmental conditions (temperature 16–20 °C; relative humidity, 29–54%; barometric pressure, 746–773 mmHg). Participants were instructed to avoid strenuous exercise in the 48 h preceding the trial, and arrive at least 2 h post-prandial. Upon arrival at the laboratory, stature and sitting height were measured with a stadiometer (SECA GmbH & Co., Hamburg, Germany) to the nearest 0.01 m, and maturity offset was predicted for each participant using published formulae (Moore et al. 2015). Body mass was recorded with digital scales (MPMS-230, Marsden Weighing Group, Oxfordshire, UK) to the nearest 0.1 kg.

All exercise testing was performed on the same motorised treadmill (HP Cosmos Pulsar 4.0, Cosmos Sports & Medical GmbH, Munich, Germany). Throughout the testing, participants breathed through a low-dead space mask to monitor expired air via an open-circuit metabolic cart (Oxycon Pro, Erich Jaeger GmbH, Germany). Before testing, gas analysers were calibrated with known gas concentrations (16% O_2_; 5% CO_2_) and ventilation measurement with a 3-L syringe. Participants completed a standardized warm-up involving a 5-min run at 2 km h^−1^ slower than the pre-determined start speed for their exercise test. Each test involved a sub-maximal discontinuous incremental test followed by an incremental continuous test to volitional exhaustion. The sub-maximal test involved 5–7 × 3-min stages interspersed with 30-s rest periods for extraction of a 20 µL capillary blood sample. The sample was immediately haemolysed in a micro-test tube and tested for blood lactate (Biosen C-Line, EKF Diagnostic, Ebendorfer Chaussee 3, Germany). The start speed of the test was determined using participants’ best race times and published recommendations (Jones 2006). Speed was increased by 1 km h^−1^ every stage until lactate turnpoint (LTP) had been surpassed, which was defined as the speed before a rise of > 1 mMol L^−1^ compared to the previous stage. The gradient of the treadmill remained at 1% throughout the sub-maximal test (Jones and Doust 1996).

Following a 5-min passive recovery, participants ran continuously at the speed corresponding to their LTP (sLTP). At the end of each minute, the treadmill gradient was increased by 1% until volitional exhaustion was reached (typically 6–8 min).

### Physiological measures

#### Sub-maximal measures

Prior to analysis of expired gases, data were filtered to remove any values that were deemed to represent errant breaths (Lamarra et al. 1987). The absence of a $\dot{V}$O_2_ slow component was verified by calculating the difference between the first 30 s of the final minute and the last 30 s. A difference less than the minimal detectable change (MDC), calculated as standard error of the mean × 1.96 × $\surd$2, confirmed a $\dot{V}$O_2_ steady state had been achieved. The final 60 s of each submaximal stage was averaged for $\dot{V}$O_2_, volume of expired CO_2_ and RER. Updated non-protein quotient equations (Peronnet and Massicotte 1991) and RER values were used to estimate $\dot{E}$_aero_ at each speed. Values for the sLTP and the three speeds prior (sLTP − 1 km^.^h^−1^, sLTP − 2 km^.^h^−1^, sLTP − 3 km^.^h^−1^) were used as the measure of RE, and quantified as both $\dot{O}$_aero_ and $\dot{E}$_aero_. For each of the four submaximal speeds, the intensity relative to each participants $\dot{V}$O_2_max or $\dot{E}$_aero_max was calculated and expressed as a percentage. Fractional utilization at the speed corresponding to lactate threshold (sLT) was also quantified. sLT was defined as the final speed prior to an initial rise (≥ 0.2 mmol L^−1^) of blood lactate from baseline, which is greater than the typical error of measurement at this speed in a similar cohort (10).

#### Maximal measures

The highest average $\dot{V}$O_2_ attained within a 30-s period during the maximal test was defined as a participant’s $\dot{V}$O_2_max. Confirmation that $\dot{V}$O_2_max had been attained was identified using an objective procedure (Midgley et al. 2009). A predicted $\dot{V}$O_2_max was calculated using the linear regression line obtained from the $\dot{V}$O_2_ data between + 2 min following the start of the test and − 2 min prior to exhaustion. A plateau was confirmed if the difference between the predicted and recorded $\dot{V}$O_2_max values was greater than 0.5 times the regression gradient. $\dot{E}$_aero_max was obtained by multiplying the $\dot{V}$O_2_max value (expressed in mL min^−1^) by 21.745 joules. s$\dot{V}$O_2_max was identified by substitution of values into the linear regression equation representing the $\dot{V}$O_2_–speed relationship from the sub-maximal running assessment.

### Allometric scaling

It is well recognised that when expressing variables relative to body size, the use of ratio scaling is inappropriate (Tanner 1949). Consequently, when comparing youth performers or individuals of different sexes, allometric expression of variables is more appropriate (Curran-Everett 2013). To obtain allometrically scaled exponents for the population under investigation, body mass and $\dot{V}$O_2_ data were log transformed and linear regression lines compared for males and females using an analysis of co-variance (ANCOVA) model. Results revealed homogeneity of regression for the slopes of all variables, thus a common scaling exponent was derived on the logarithmic transformed data sets. The appropriateness of the power function was confirmed using an absence of relationships derived from the linear regression correlations between body mass and $\dot{V}$O_2_ scaled values.

### Performance measures

Participant’s best times over 0.8, 1.5 and 3 km during competitive track races, within 60 days (41 ± 17 days) of laboratory testing, were converted to running speed as an index of performance.

### Statistical analysis

Data were analysed with IBM SPSS Statistics (v24) and values are displayed as mean ± standard deviation (SD). A *p* value of < 0.05 was used to denote statistical significance. Normality in distribution of the dependent variables was assessed using the Shapiro–Wilk statistic and homogeneity of variance with Levene’s test. Data from a number of race distances did not conform to this assumption, thus running speeds were log-transformed prior to further analysis. Normality associated with the standardized residual errors was assessed using probability plots and confirmed objectively using the standard residual statistic. Homoscedasticity was assessed using scatterplots of the residual errors and predicted values. Several variables displayed multicollinearity, defined as an *r* value > 0.7. Speed at LTP and s$\dot{V}$O_2_max were, therefore, analysed as separate independent variables with one-tailed Pearson correlation tests. For each race distance, multiple linear regression models were used to examine the combined influence of predictors expressed in terms of the $\dot{O}$_aero_ measures, and predictors quantified as $\dot{E}$_aero_. Zero-order correlation statistics were used to interpret the relationship with each variable in the model. To compare the correlation statistics for $\dot{O}$_aero_-related measures against those expressed as $\dot{E}$_aero_, a 95% confidence interval (CI) was calculated for each result. Correlation coefficients were interpreted as ≤ 0.30 negligible correlation, 0.31–0.50 low correlation, 0.51–0.70 moderate correlation, 0.71–0.90 high correlation, > 0.90 very high correlation (Hinkle et al. 2003).

A one-way repeated measures analysis of variance (ANOVA) was performed to evaluate the differences between $\dot{O}$_aero_ and $\dot{E}$_aero_ across four relative running speeds. Differences between %$\dot{V}$O_2_max and %$\dot{E}$_aero_max were assessed using a two-way (measure × speed) ANOVA and the differences between individual relative speeds was analysed using a one-way ANOVA. Bonferroni post hoc adjustments were used to detect any significant differences between individual speeds or measures.

## Results

Performance times for males and females are shown in Table 2. Allometric scaling revealed exponents that approximated three-quarters for $\dot{V}$O_2_ at each speed [sLTP: *b* = 0.77 (95% CI 0.54–0.99), sLTP − 1 km h^−1^: *b* = 0.77 (95% CI 0.54–0.99), sLTP − 2 km h^−1^: *b* = 0.78 (95% CI 0.56–0.99), sLTP − 3 km h^−1^: *b* = 0.84 (95% CI 0.64–1.05)] and $\dot{V}$O_2_max [*b* = 0.74 (95% CI 0.48–1.00)]. Applying this power function (*b* = 0.75) revealed an absence of any significant relationship between body mass and scaled $\dot{V}$O_2_ across the intensities assessed (*r* ≤ 0.14, *p* ≥ 0.36).

**Table 2:** Performance time (mean ± standard deviation), coefficients (with 95% CI) of multiple regression, and Pearson correlations for physiological variables and running performance speed in males and females

Distance	Time (s)	Multiple regression adjusted *r*^2^		Mean running economy^A^		Fractional utilization at sLT^A^		$\dot{V}$O_2_max^A^	s$\dot{V}$O_2_max	sLTP
		$\dot{O}$_aero_	$\dot{E}$_aero_	$\dot{O}$_aero_	$\dot{E}$_aero_	%*˙V̇*O_2_max	%$\dot{E}$_aero_max			
Males										
0.8 km (*n* = 21)	120.8 ± 8.8	0.40^b^	0.39^b^	− 0.53^a^ (− 0.78 to − 0.13)	− 0.52^a^ (− 0.78 to − 0.12)	0.19 (− 0.26 to 0.57)	0.21 (− 0.25 to 0.59)	0.14 (− 0.31 to 0.55)	0.45 (− 0.01 to 0.72)	0.60^b^ (0.20 to 0.81)
1.5 km (*n* = 34)	250.8 ± 17.9	0.56^c^	0.57^c^	− 0.33 (− 0.60 to 0.01)	− 0.39^a^ (− 0.64 to − 0.06)	− 0.03 (− 0.31 to 0.36)	− 0.04 (− 0.29 to 0.38)	0.55^b^ (0.27 to 0.75)	0.75^c^ (0.55 to 0.87)	0.78^c^ (0.60 to 0.89)
3 km (*n* = 21)	539.5 ± 43.5	0.84^c^	0.85^c^	− 0.60^b^ (− 0.82 to − 0.23)	− 0.63^b^ (− 0.83 to − 0.27)	− 0.15 (− 0.52 to 0.33)	− 0.14 (− 0.50 to 0.34)	0.77^c^ (0.52 to 0.91)	0.93^c^ (0.77 to 0.97)	0.90^c^ (0.77 to 0.96)
Females										
0.8 km (*n* = 16)	136.6 ± 3.7	0.58^b^	0.44^a^	− 0.66^b^ (− 0.87 to − 0.25)	− 0.40 (− 0.74 to 0.13)	0.02 (− 0.49 to 0.50)	0.07 (− 0.43 to 0.55)	0.52^a^ (0.04 to 0.81)	− 0.01 (− 0.50 to 0.49)	0.22 (− 0.31 to 0.64)
1.5 km (*n* = 22)	281.4 ± 11.8	0.10	0.11	− 0.34 (− 0.68 to 0.08)	− 0.37 (− 0.69 to 0.05)	− 0.29 (− 0.64 to 0.15)	− 0.33 (− 0.66 to 0.11)	0.36 (− 0.08 to 0.68)	0.42 (0 to 0.72)	0.55^a^ (0.17 to 0.79)
3 km (*n* = 16)	622.0 ± 36.3	0.79^c^	0.73^c^	− 0.57^a^ (− 0.83 to − 0.11)	− 0.59^a^ (− 0.84 to − 0.14)	− 0.64^b^ (− 0.86 to − 0.22)	− 0.66^b^ (− 0.87 to − 0.25)	0.77^c^ (0.45 to 0.92)	0.84^c^ (0.59 to 0.94)	0.85^c^ (0.62 to 0.95)

$\dot{O}$_*aero*_ aerobic oxygen cost, $\dot{E}$_*aero*_ aerobic energy cost, $\dot{V}$*O*_*2*_*max* maximal oxygen uptake, $\dot{E}$_*aero*_*max* maximal aerobic energy expenditure, $s{\dot{V}}O$_*2*_*max* speed at $\dot{V}$O_2_max, *sLTP* speed at lactate turn point

^a^*p* < 0.05, ^b^*p* < 0.01, ^c^*p* < 0.001

^A^Variables used in multiple regression analysis

Table 2 shows a high level of similarity between the correlation coefficients for the two methods used to quantify aerobic energy expenditure. Multiple-regression analysis revealed that the independent variables of mean RE, fractional utilization at sLT, and $\dot{V}$O_2_max, accounted for > 80% and > 70% of the variance in 3 km performance in males and females, respectively (*p* < 0.001). These three variables were also significant predictors of 0.8 km (*p* < 0.01) and 1.5 km (*p* < 0.001) performance in males, but were poor predictors of 1.5 km performance in females. s$\dot{V}$O_2_max and sLTP tended to correlate strongly with performance over longer distances but the relationships were weaker for 0.8 km in both sexes (Table 2).

**Fig. 1:**
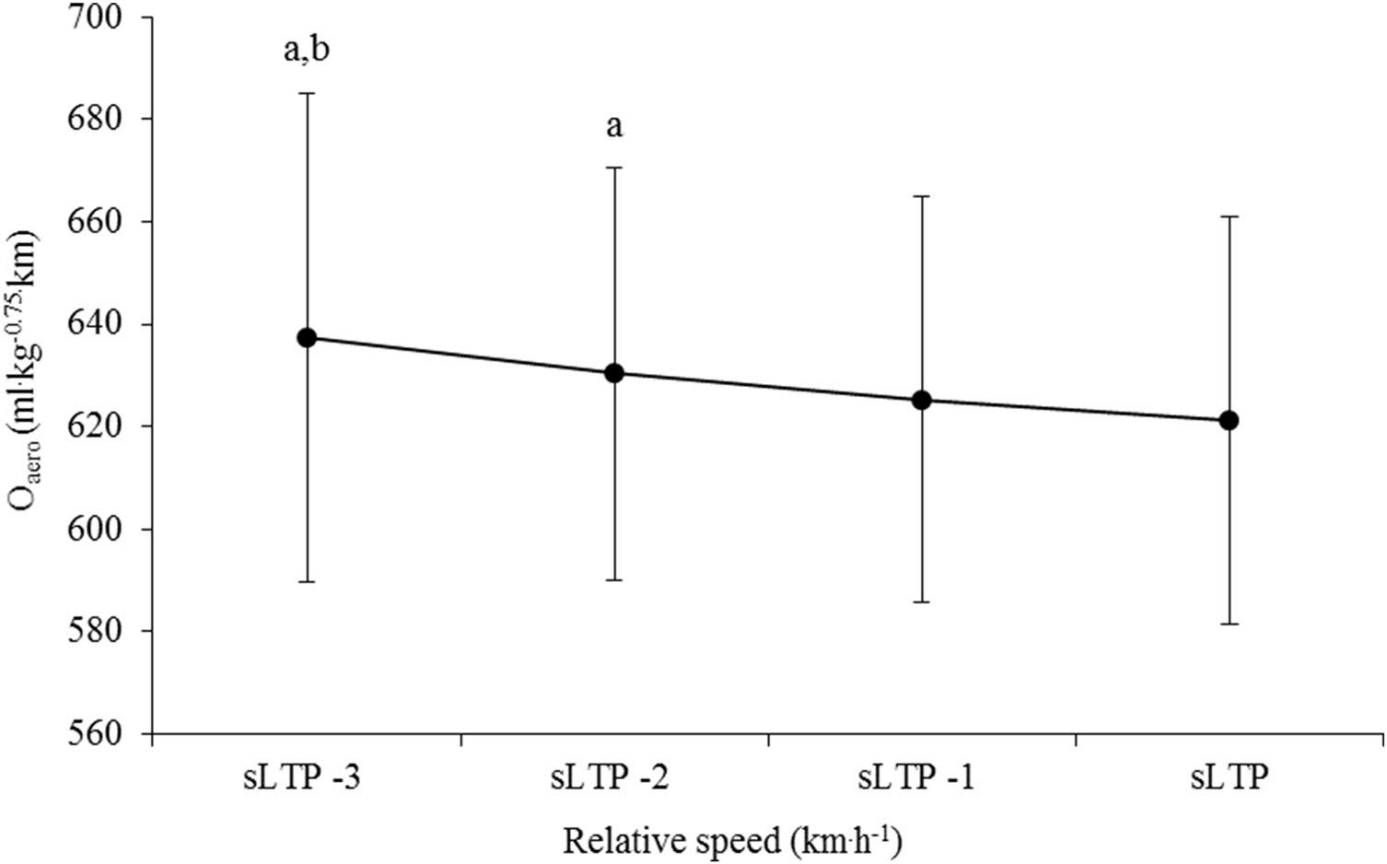
Oxygen cost ($\dot{O}$_aero_) for speed at lactate turnpoint (LTP) and the three speeds prior (*n* = 56). ^a^Significantly different from speed at LTP (*p* < 0.01), ^b^significantly different from speed at LTP-1 km h^−1^ (*p* = 0.01)

**Fig. 2:**
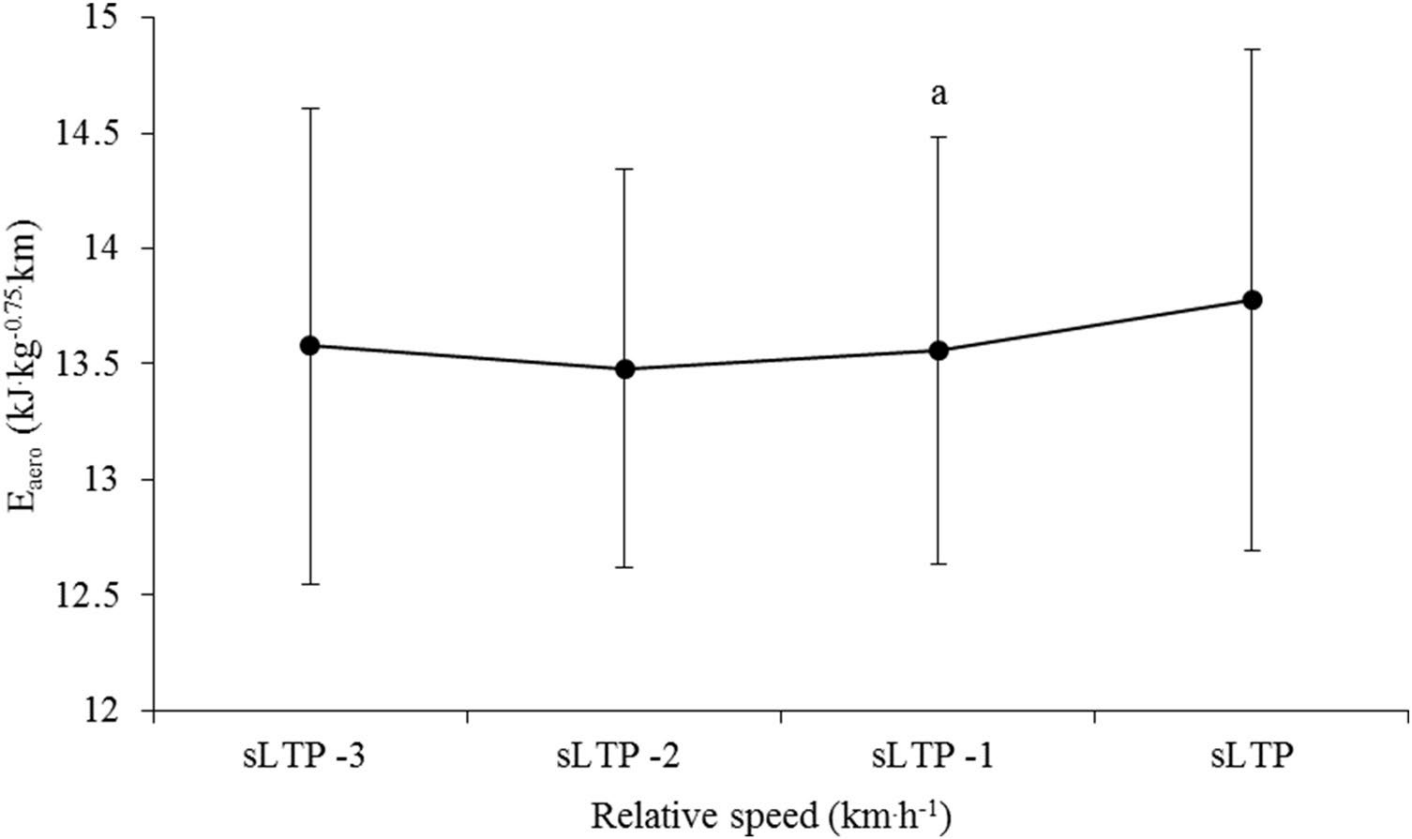
Aerobic energy cost ($\dot{E}$_aero_) for speed lactate turnpoint (LTP) and the three speeds prior (*n* = 56). ^a^Significantly different from speed at LTP (*p* = 0.02)

ANOVA revealed a significant decrease in $\dot{O}$_aero_ as running speed increased (*F* = 11.59, *p* < 0.001, Fig. 1). Post hoc analysis revealed significant differences between $\dot{O}$_aero_ at a number of individual speeds (Fig. 1) and two other comparisons (sLTP vs sLTP − 1 km h^−1^, sLTP − 1 km h^−1^ vs sLTP − 2 km h^−1^) approached significance (*p* = 0.07). A significant effect of running speed was also noted for $\dot{E}$_aero_ (*F* = 4.74, *p* = 0.015, Fig. 2). Post hoc inspection identified a difference between sLTP and sLTP − 1 km h^−1^ (*p* = 0.02); however, the difference between sLTP and sLTP − 2 km h^−1^ was close to the threshold of significance (*p* = 0.06).

A significant main effect between the slopes of the lines was detected for %$\dot{V}$O_2_max and %$\dot{E}$_aero_max when plotted against relative running speed (*F* = 5.38, *p* = 0.021); however, there was an absence of an interaction effect (measure × speed; *F* = 0.29, *p* = 0.834). One-way ANOVA analysis was also not able to locate any difference between measures at each relative speed.

## Discussion

The primary aim of this study was to examine the relationship between race performances and several important aerobic variables quantified as both $\dot{E}$_aero_ and $\dot{O}$_aero_ in adolescent middle-distance runners. Results indicate that $\dot{E}$_aero_ does not provide a greater level of criterion validity compared to $\dot{O}$_aero_-based measurements in this age group for the middle-distance running events. The study also aimed to investigate the validity of $\dot{O}$_aero_ and $\dot{E}$_aero_ as a means of quantifying RE. Results showed differences in the manner $\dot{O}$_aero_ and $\dot{E}$_aero_ change with increasing running speed, with $\dot{O}$_aero_ displaying a decrease and $\dot{E}$_aero_ a curvilinear response. A further finding was that the relationship between relative running speed and the fraction of $\dot{V}$O_2_max or $\dot{E}$_aero_max that is accessed also appears to differ, with the difference being greater at lower intensities. These findings provide new insight into the ongoing debate surrounding the most appropriate method of expressing aerobic fitness parameters, which are typically used to evaluate performance, health status and monitor improvement.

Results of the multiple regression analysis show that using $\dot{E}$_aero_ to quantify RE and fractional utilization, instead of traditional $\dot{O}$_aero_, provides no additional value in the prediction of middle-distance running performance in adolescents. To alter the strength of the relationship between $\dot{E}$_aero_-based determinants and performance, a high-level of inter-individual variability in substrate utilization is required. This was not apparent as coefficient of variation (SD/mean) for the RER values at each relative speed was ~ 4%. The participants used in this study possessed somewhat homogenous physiological characteristics (Table 1), thus future research could investigate a more heterogeneous sample of runners, who are likely to differ more substantially in terms of their consumption of substrates at the same relative speeds. Similarly, a relatively small range of running speeds (sLTP to sLTP − 3 km h^−1^) was examined in the present study and measurement stages were relatively short (3 min), which resulted in mean RER values > 0.9. A larger range of speeds and longer sampling duration would produce lower RER values (Van Loon et al. 2001) and may have generated more substantial between-participant variability as maximal rates of lipid oxidation rates are known to occur at ~ 65% $\dot{V}$O_2_max but is dependent upon training status (Achten and Jeukendrup 2004). This would, therefore, alter the predictive power of variables quantified in $\dot{E}$_aero_ units. Nevertheless, it is also questionable that slower running speeds would correlate well with middle-distance performance given the large discrepancy between low-intensity running and middle-distance race speed. Although $\dot{E}$_aero_ also accounts for the energy yield associated with work performed during sub-maximal exercise compared to $\dot{O}$_aero_ (Shaw et al. 2014), there are numerous other factors that also govern these parameters, including use of stretch-shortening cycle mechanisms, muscle activation in the musculotendinous unit, running kinematics and anthropometric factors (Barnes and Kilding 2015). Thus, it may also be the case that the additional metabolic insight, which $\dot{E}$_aero_ provides, is insufficient to alter the predictive capacity of these aerobic parameters on performance. Within study designs that assess participants at more than one point in time, expressing RE as $\dot{E}$_aero_, rather than $\dot{O}$_aero_, is likely to provide the most scientifically robust metric (Blagrove et al. 2017; Shaw et al. 2014).

To the author’s knowledge, this is the first study to apply a multiple regression model to middle-distance performance in adolescent runners, using acknowledged aerobic determinants of performance (Bassett and Howley 2000; Ingham et al. 2008). Results showed that a high level (~ 80%, *p* < 0.001) of inter-individual variability in male and female 3 km performance could be explained by RE, fractional utilization at sLT and $\dot{V}$O_2_max (Table 2). Moreover, ~ 40% (*p* < 0.01) of the 0.8 km performance variability and 56% (*p* < 0.001) of male 1.5 km performance could be explained using these same variables. Surprisingly, this regression model could only predict a small (10%) amount of the variability in female 1.5 km performance. This may be due to the homogeneity of the performance times in the female (5%) compared to the male sample (7%) over 1.5 km.

Previous studies have shown the importance of $\dot{V}$O_2_max for middle-distance (1.5 km and 3 km) performance in children and adolescent (Abe et al. 1998; Mahon et al. 1996; Unnithan et al. 1995), which is largely confirmed by the results of this investigation (*r* = 0.55–0.77, *p* < 0.01). Based upon the non-overlap of the 95% CI with the correlation coefficients, it is also apparent that $\dot{V}$O_2_max becomes more important as a determinant of performance as race duration increases, which is in agreement with previous findings in adolescent (Almarwaey et al. 2003) and adult runners (Ingham et al. 2008; Padilla et al. 1992). It is likely that this pattern in results reflects the increasing proportion of $\dot{V}$O_2_max that is attained as race duration increases in middle-distance events (Brandon 1995). This is also the case for s$\dot{V}$O_2_max and sLTP, both of which show high (*r* > 0.84, *p* < 0.001) correlations with 3 km performance in males and females but weaker correlations at the shorter distances (Table 2). RE, as an independent factor, is not thought to be important for middle-distance running performance (Ingham et al. 2008) despite several studies observing significant relationships in young runners (Almarwaey et al. 2003; Mayers and Gutin 1979; Unnithan et al. 1995). When RE was expressed as $\dot{E}$_aero_, it generally showed low–moderate negative relationships (*r* = − 0.37 to − 0.63) with performance, which did not differ across race distances (Table 2). Relationships were significant for male participants across all distances (*p* < 0.05) and females only at 3 km (*r* = − 0.57, *p* < 0.05), which is in agreement with the previous findings (Almarwaey et al. 2003). In adolescent middle-distance running, it, therefore, appears that RE influences race performance, but explains a relatively small proportion of inter-individual variability. It is possible that participants who have a low $\dot{V}$O_2_max compensate by possessing better RE (Cunningham 1990). This may explain the low relationship (*r* = 0.14) between $\dot{V}$O_2_max and 0.8 km performance in males but moderate relationship (*r* = − 0.52, *p* < 0.05) between $\dot{E}$_aero_ and performance over this distance.

Results demonstrate that the RE–speed relationship differed depending upon the strategy used to quantify RE. When expressed as $\dot{O}$_aero_, running became less metabolically expensive as a function of speed (*F* = 11.59, *p* < 0.001, Fig. 1), which is in agreement with previous findings (Iaia et al. 2009) but in contrast to others who have shown no change (Fletcher et al. 2009; Shaw et al. 2014) or an increase (Fletcher et al. 2013) in $\dot{O}$_aero_ as speed increases. This discrepancy between findings is likely due to the range of speeds examined in each study and the training status of participants. Similar to the study by Iaia et al. (2009), the speeds selected in the present study represent the upper end of the range over which RE can be measured with high validity (≤ LTP, RER < 1.0), whereas others have utilized a lower range of relative intensities (Fletcher et al. 2009; Shaw et al. 2014). Furthermore, previous studies used highly trained runners (Fletcher et al. 2009; Shaw et al. 2014), who were assessed at faster absolute speeds compared to the young runners recruited in the present study. When quantified as $\dot{O}$_aero_ (per unit distance), a faster range of absolute speeds tends to produce a flatter relationship compared to oxygen cost at slower absolute speeds.

**Fig. 3:**
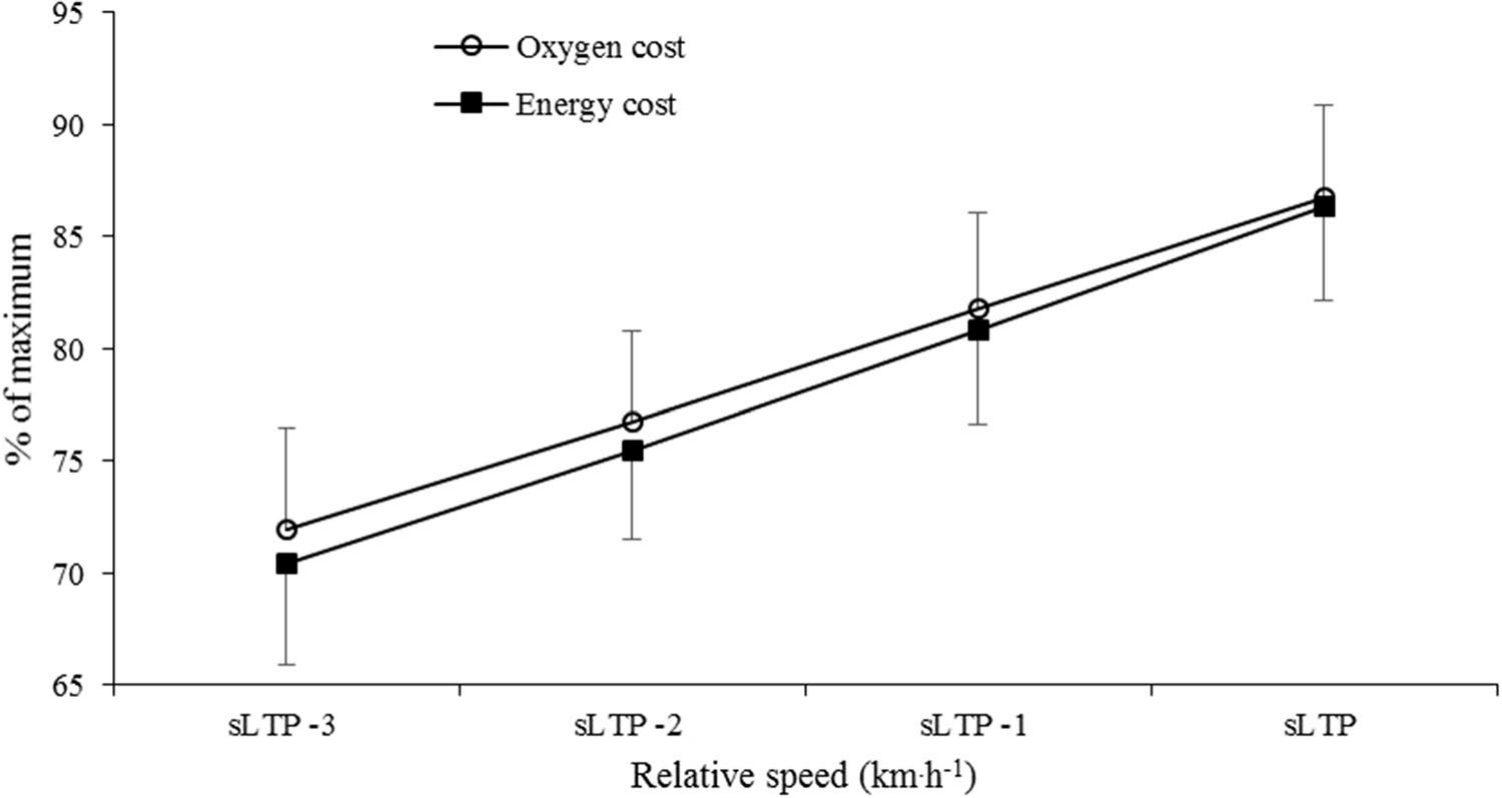
Percentage utilization of maximum oxygen uptake and maximum aerobic energy expenditure across four sub-maximal relative speeds (*n* = 56). *sLTP* speed at lactate turnpoint

Conversely, when RE was quantified as $\dot{E}$_aero_, a subtle ‘U-shaped’ profile was apparent across the range of speeds (Fig. 3), with a significant difference noted between sLTP and sLTP − 1 km h^−1^ (*p* = 0.02) and a near-significant difference between sLTP and sLTP − 2 km h^−1^ (*p* = 0.06). A curvilinear relationship between $\dot{E}$_aero_ and speed has been observed in a number of studies (Black et al. 2018; Rathkey and Wall‐Scheffler 2017; Steudel-Numbers and Wall-Scheffler 2009; Willcockson and Wall-Scheffler 2012), with the nadir representing the most economical running speed. The least energetically expensive speed (at sLTP − 2 km h^−1^) in the present study was 13.4 ± 1.7 km h^−1^, which is similar to the 13 km h^−1^ (Black et al. 2018) and 12.6 km h^−1^ (Steudel-Numbers and Wall-Scheffler 2009; Willcockson and Wall-Scheffler 2012) reported previously in similarly trained participants. It is likely that other studies that have observed linear $\dot{E}$_aero_–speed relationships have used a range of speeds that did not capture the lowest point of the curve (Fletcher et al. 2009; Shaw et al. 2014) or used a lesser trained group of runners (Black et al. 2018).

Crucially, the trend for an increase in $\dot{E}$_aero_ with faster speeds above sLTP − 2 kmh^−1^ is in opposition to the relationship demonstrated between $\dot{O}$_aero_ and running speed. $\dot{E}$_aero_ represents a theoretically more valid measure of RE as it estimates actual energy turnover, whereas $\dot{O}$_aero_ is simply a measure of the $\dot{V}$O_2_ per unit of running distance. The increase in $\dot{E}$_aero_ as speed progressed from sLTP − 2 km h^−1^ towards sLTP, therefore, reflects the increase in RER value, indicating an increased reliance on carbohydrate as an energy source. As running speed increases, joint angular velocities are greater and ground contact time reduces, which requires a greater reliance on metabolically inefficient type II muscle fibres (Fletcher and MacIntosh 2017). An increased recruitment of high threshold motor units is likely to be the mechanism that drives the rate of carbohydrate utilization, and as the energy yield from carbohydrates per mole of O_2_ is also greater than lipids (Jeukendrup and Wallis 2005), this generates higher $\dot{E}$_aero_ at faster speeds. It is, therefore, recommended that $\dot{E}$_aero_ should be used as a measure of RE as this provides a more valid indicator of the metabolic demand of running compared to $\dot{O}$_aero_.

Exercise intensity is often prescribed relative to an individual’s $\dot{V}$O_2_max, thus expression of relative aerobic intensity as a percentage of $\dot{E}$_aero_max would be more meaningful. However, prescribing exercise intensity on either basis has been criticised due to the heterogeneity at which other important exercise thresholds (critical speed, anaerobic threshold, lactate threshold) occur (Baldwin et al. 2000; Scharhag-Rosenberger et al. 2010). Therefore, an intensity expressed relative to either $\dot{V}$O_2_max or $\dot{E}$_aero_max may represent a severe intensity (relative to critical power) in one individual but provide a steady-state condition for another individual. We attempted to account for this in the present study by comparing the relationship between %$\dot{V}$O_2_max or %$\dot{E}$_aero_max across a range of running speeds expressed relative to each individual’s sLTP. A significant main effect method of measurement (%$\dot{V}$O_2_max versus %$\dot{E}$_aero_max) was detected (*F* = 5.38, *p* = 0.021); however, no differences were identified at individual relative speeds. The divergent nature of the gradients (Fig. 3) as relative intensity decreases suggests that at slower relative speeds, the use of %$\dot{E}$_aero_max becomes more important. Therefore, it is recommended that if exercise is prescribed based on maximal aerobic values, intensity is expressed as a fraction of $\dot{E}$_aero_max, rather than $\dot{V}$O_2_max. However, a superior method for prescribing running intensity is to base calculations on sLTP (or a similar objective metabolic threshold), which would reduce inter-individual differences in relative intensity, thus providing a more valid strategy.

This study has several limitations that should be acknowledged. First, physiological testing predominantly took place during the pre-season or early competitive racing period, with the duration between a participants’ race performance and laboratory testing typically 3–8 weeks. Although every attempt was made to minimize this time gap, small changes in the physiological profile of participants cannot be discounted, which may have influenced the results. Second, participants performed laboratory testing 2 h post-prandial; however, it is less certain whether this requirement was adhered to prior to races. Participants possessed ≥ 2 years’ racing experience; therefore, it is unlikely that subtle differences in pre-race routine confound the results to a large extent. Third, middle-distance running performance is limited by anaerobic factors, in addition to the aerobic determinants measured in this study (Thompson 2017). These anaerobic variables were not quantified in this investigation and are likely to explain a large proportion of the variability in performance currently unaccounted for in the regression models. Moreover, investigating the determinants of longer race distances (≥ 5 km), which have a greater reliance on aerobic sources of energy, would also have been of interest in this age group.

## Conclusions

Expression of RE and fractional utilization in terms of $\dot{E}$_aero_ rather than $\dot{O}$_aero_ does not appear to alter the ability of these determinants to predict middle-distance running performance in adolescents. RE, fractional utilization at sLT and $\dot{V}$O_2_max accounted for approximately 80% of the variability in 3 km performance in adolescent males and females. These variables could explain less (40–60%) of the variation in performance over shorter race distances and very little over 1.5 km in females. s$\dot{V}$O_2_max and sLTP were confirmed as other important indicators of middle-distance performance in adolescent runners with the strength of relationships tending to be greater over longer distances. Results also indicate markedly different profiles in the $\dot{O}$_aero_–speed response compared to $\dot{E}$_aero_–speed relationship. It is recommended that RE is quantified in $\dot{E}$_aero_ units, which provides a more valid reflection of the metabolic demand of running across a range of speeds. Finally, there were differences observed in the slope of the relationships between running speed and the proportion of $\dot{V}$O_2_max or $\dot{E}$_aero_max utilized at each speed, suggesting this should be accounted for if prescribing exercise intensity using this method.

## Abbreviations

ANCOVA: Analysis of co-variance
ANOVA: Analysis of variance
CI: Confidence interval
$\dot{E}$_aero_: Aerobic energy cost
$\dot{E}$_aero_max: Maximal aerobic energy expenditure
LTP: Lactate turnpoint
MDC: Minimal detectable change
$$\dot{O}$$_aero_: Oxygen cost
RE: Running economy
RER: Respiratory exchange ratio
SD: Standard deviation
sLT: Speed corresponding to lactate threshold
sLTP: Speed corresponding to lactate turnpoint
s$$\dot{V}$$O_2_max: Speed at $$\dot{V}$$O_2max_
$$\dot{V}$$O_2_: Oxygen uptake
$\dot{V}$O_2_max: Maximal oxygen uptake
